# Paternal depression in the postpartum year and children’s behaviors at age 5 in an urban U.S. birth cohort

**DOI:** 10.1371/journal.pone.0300018

**Published:** 2024-04-18

**Authors:** Kristine Schmitz, Manuel E. Jimenez, Hope Corman, Kelly Noonan, Nancy E. Reichman

**Affiliations:** 1 Department of Pediatrics, Division of Population Health, Quality and Implementation, Robert Wood Johnson Medical School, Rutgers University, New Brunswick, New Jersey, United States of America; 2 Child Health Institute of New Jersey, Rutgers University, New Brunswick, New Jersey, United States of America; 3 Department of Economics, Rider University, Lawrenceville, New Jersey, United States of America; 4 National Bureau of Economic Research, New York, New York, United States of America; 5 Department of Economics, Princeton University, Princeton, New Jersey, United States of America; Virginia Tech Carilion School of Medicine, UNITED STATES

## Abstract

**Objective:**

To investigate associations between postpartum depression in fathers and children’s behaviors at age 5 in a national high-risk U.S. sample.

**Study design:**

A secondary data analysis of 1,796 children in a national birth cohort study that oversampled non-marital births was conducted. Paternal depression was assessed 1 year after the child was born and children’s behaviors were assessed by their primary caregivers when the children were 5 years old. Unadjusted and adjusted negative binomial regression models of associations between paternal depression and child behavior scores and logistic regression models of associations between paternal depression and high scores (at least 1.5 or 2.0 standard deviations above the mean) were estimated.

**Results:**

In negative binomial regression models that adjusted for child, paternal, and family characteristics and maternal depression, paternal depression was associated with a 17% higher total externalizing behavior score (Incidence Rate Ratio (IRR): 1.17; 95% Confidence Interval (CI): 1.07–1.27), a 17% higher aggressive subscale score (IRR: 1.17; 95% CI: 1.08–1.27), and an 18% higher delinquent subscale score (IRR: 1.18; 95% CI: 1.03–1.35). In adjusted logistic regression models for scores ≥2.0 standard deviations above the mean, paternal depression was associated with high total externalizing scores (e.g., Odds Ratio (OR): 3.09; 95% CI: 1.77–5.41), high aggressive behavior scores (OR: 2.40; 95% CI: 1.30–4.43), and high delinquent behavior scores (OR: 2.08; 95% CI: 1.01–4.27). There were suggestive but non-robust associations between paternal depression and attention problems and no associations between paternal depression and internalizing behaviors or social problems.

**Conclusion:**

Fathers’ depression at age 1 was associated with children’s externalizing behaviors at age 5, an important developmental stage when children transition to school. These findings suggest a need to identify and support fathers with depressive symptoms to promote optimal child development.

## Introduction

Maternal depression has been linked to adverse child developmental and behavioral outcomes, including depression [1, 2], poor peer interactions [3], externalizing and internalizing disorders [4], emotional dysregulation [4], and poor school achievement [5]. Relatively few studies have investigated links between paternal depression and child outcomes, and most of those have been based on countries outside of the U.S., which has unique demographic, healthcare, and policy contexts. Moreover, the two U.S.-based studies that were based on nationally representative samples focused on families in which the father lived in the child’s household [6, 7], despite the realities that: (1) Many births in the U.S. take place outside of marriage; for example, in 2020, 40.5% of all U.S. births were to unmarried parents [8]. (2) Parents in the U.S. who are in non-marital cohabiting unions when their children are born have high rates of relationship dissolution; e.g., only about one third of children in an urban U.S. birth cohort in 1998–2000 who born out of wedlock lived with their fathers at age 5 [9]. (3) Most unmarried fathers in large U.S. cities, including those who have never been married to or lived with their children’s mothers, spend time with and engage in activities with their young children [10]. (4) Non-marital childbearing is strongly associated with low socioeconomic status in the U.S. [11].

Paternal postpartum depression has been acknowledged as a phenomenon related to having had a child during the past year and has received increasing attention from the medical and research communities [12, 13]. Rates of perinatal depression among fathers range from 2 to 25% [14]. Fathers in the U.S. who are unmarried are at particular risk for depression because they tend to have low earnings [15] and low rates of health insurance and other public supports [16]; indeed, they have higher rates of depression than both mothers and married fathers [17]. Elucidating links between exposure to paternal depression and child developmental outcomes at key developmental periods in a U.S. population that includes non-residential fathers can inform interventions that identify and support families and enhance child development.

Emerging research from around the world has found associations between depression in fathers and adverse behavioral, developmental, social, and emotional outcomes of children and adolescents [18–22], but many of the studies were based on small non-representative samples. Population-based studies have found associations between paternal depression and children’s externalizing behaviors, such as oppositional defiant disorder, hyperactivity, and conduct disorder [23–27]; internalizing behaviors, such as depression, anxiety, or impaired peer and social interactions [6, 24, 26, 28]; and general measures of behavioral problems [7], but many did not consider prospective associations or focus on specific developmentally important ages.

To our knowledge, no national U.S.-based studies have considered prospective associations between exposures to paternal depression and children’s subsequent behavioral outcomes at key developmental stages. In this study, we investigated associations between depression in fathers of infants and their children’s behavioral and social problems at age 5 in a national U.S. birth cohort that over-sampled non-marital births, which in the U.S. are a policy-relevant population. We focused on paternal depression during the postpartum year, a critical period for parenting [13, 29] and child development [14, 30], and children’s behaviors at the age when they typically transition to school and behaviors may manifest or become more apparent. Behavior problems at this stage are associated with later academic difficulties [31]. In turn, poor social-emotional development and behavioral problems in school-aged children can lead to adverse educational and mental health outcomes, problematic peer relationships, and risky health behaviors [32–35]. We hypothesized that paternal depression during the postpartum year is associated with children’s behavioral problems at age 5, when they typically transition into school, even when controlling for baseline child, paternal, and family characteristics as well as maternal depression during the postpartum year.

## Methods

### Data

We use data from the Future of Families and Child Wellbeing (FFCWB) study, a national birth cohort study that randomly sampled births in 75 hospitals in 20 large U.S. cities in 1998–2000. This cohort was population-based because it used a probability sample of cities, hospital, and births within hospitals. Non-marital births were oversampled by design in the original study; that is, marital births were no longer included when hospital-specific quotas were reached. The reason for the oversampling was that unmarried parents, particularly fathers, had been largely neglected in national U.S. datasets. About three-fourths of the mothers in the study were unmarried [36, 37]. Because non-marital childbearing is strongly associated with poverty in the U.S. [11], as is racial/ethnic minority status [38], the FFCWB sample had high fractions of poor, Black, and Hispanic parents.

Face-to-face interviews were conducted with 4,898 mothers while still in the hospital after the child’s birth and 3,830 of the children’s fathers. One year later, 4,457 mothers (91% of baseline sample) and 3,132 fathers (82% of baseline) were re-interviewed. When the child was 5 years old, 4,295 primary caregivers (88% of baseline mothers) were re-interviewed. The primary caregiver at age 5 was the child’s mother in about 98% of the cases. The data are free to download from Princeton University’s Office of Population Research data archive (at https://ffcws.princeton.edu/documentation).

**Fig 1:**
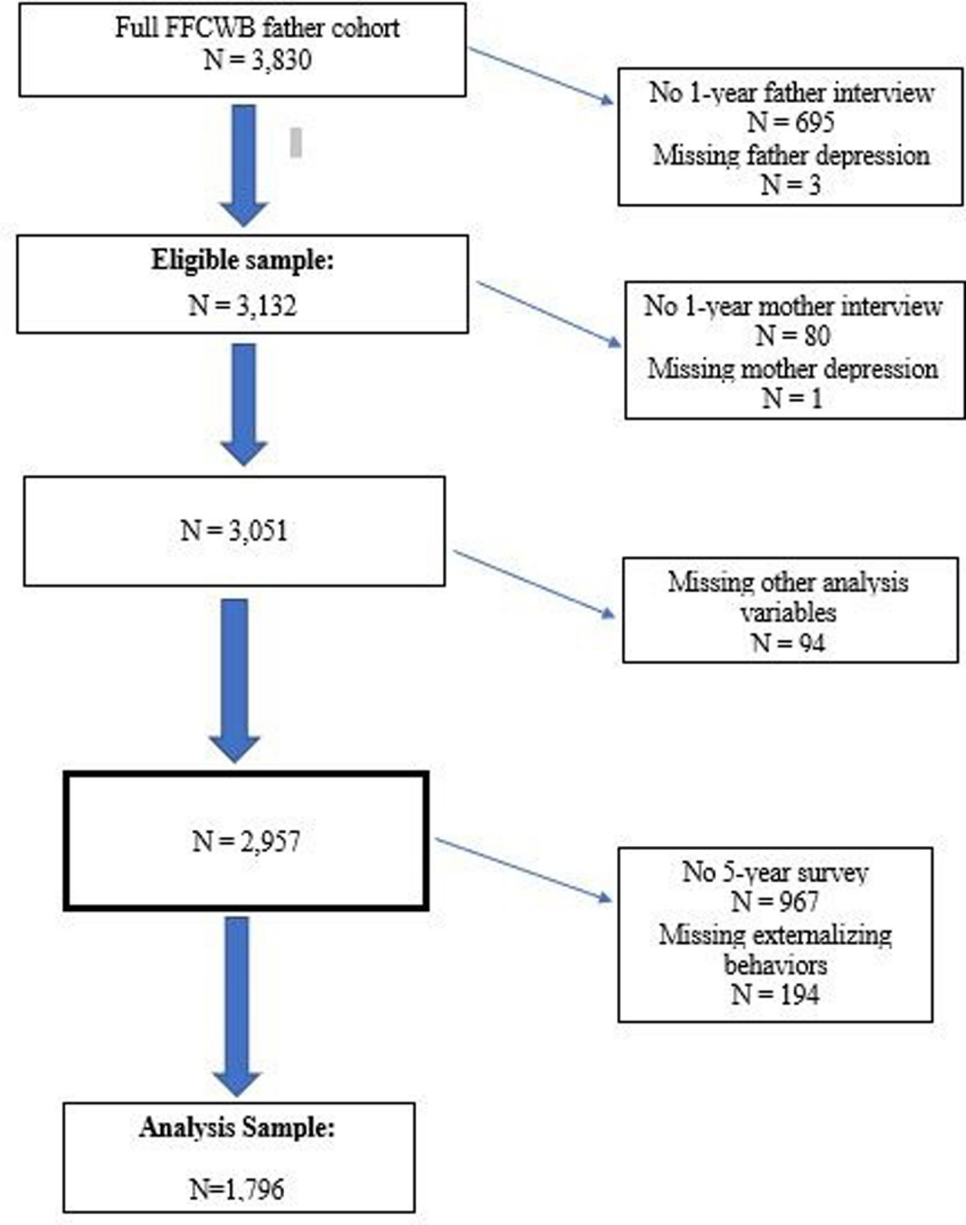
Derivation of analysis sample.

Of the 3,132 fathers who completed the 1-year survey, which included a depression screener, 81 had missing information on maternal depression at the 1-year survey and another 94 had missing data on other analysis variables (Fig 1). Of the remaining 2,957, the primary caregiver did not complete a survey at 5 years in 967 cases. Of the cases that did have 5-year primary caregiver survey data, incomplete responses to questions pertaining to the child behavior scales resulted in slightly different analysis samples across outcomes.

### Measures

#### Paternal depression

Paternal depression was assessed using the World Health Organization’s Composite International Diagnostic Interview Short Form (CIDI-SF) Version 1.0, an instrument based on the Diagnostic and Statistical Manual of Mental Disorders–Fourth Edition (DSM-IV) criteria. The CIDI-SF is a widely used screening scale that has been shown to have an accuracy rate of 93% for a major depressive episode [39]. Our main measure of paternal depression at 1 year, an indicator for whether the father met diagnostic criteria for major depression in the past 12 months, was defined as having experienced three or more symptoms of dysphoria or anhedonia for most of the day on all or most days in any 2-week period during the past year. The symptoms of dysphoria or anhedonia include feeling sad, blue, or depressed; losing interest in pleasurable activities; fatigue; trouble sleeping; trouble concentrating; feelings of worthlessness; and death ideation. As an alternative, we also used a less stringent measure—having experienced three or more symptoms for at least half of the day on all or most days during any 2-week period in the past year—in supplementary analyses. Both measures have been validated [40]. The precise coding of the measures of depression is described in the FFCWB scales documentation [36].

#### Children’s behavioral outcomes

Behaviors were assessed using responses to selected items from Achenbach’s Child Behavior (CBCL) Checklist 2–3 [41, 42] that were reported by primary caregivers when the children were 5 years old. This instrument is often used to characterize externalizing behaviors, internalizing behaviors, attention problems, and social problems in children [43]. Primary caregivers were asked to rate the extent to which their child exhibited specific behaviors, and the response for each type of behavior was coded as 0 for “not true,” 1 for “somewhat or sometimes true,” or 2 for “very true or often true.” For each scale or subscale, the responses to relevant items were summed. If there were any missing responses, the score was set to missing. Higher scores indicate more behavior problems. The questions used to create each of the scales and subscales are listed in S1 Table [42].

The total externalizing scale, based on 30 items, has a Chronbach’s alpha of 0.87 in our sample and consists of two sub-scales—aggressive and delinquent, which have alphas of 0.86 and 0.55, respectively. The total internalizing scale, based on 22 items, has an alpha of 0.77 and consists of two sub-scales—anxious and withdrawn, which have alphas of 0.71 and 0.60, respectively. The attention problems scale is based on 11 items, with an alpha of 0.68, and the social problems scale has 8 items, with an alpha of 0.43.

#### Control variables

All adjusted models controlled for the sex assigned to the child at birth and whether the child was low birth weight (< 2500 grams); paternal age, race/ethnicity, foreign-born status, and education; parents’ marital/cohabitation status; and Medicaid coverage for the delivery. Medicaid is the public health insurance program available to low-income people in the U.S., and a Medicaid-financed delivery is a proxy for poverty. All controls were assessed at baseline; the categories for race/ethnicity and education are detailed in Table 1. Because paternal depression is associated with maternal depression [12], some models further controlled for mother’s depression at 1 year, which was also assessed using the CIDI-SF.

**Table 1:** Child and parent characteristics, overall and by paternal depression at 1 year.

		Paternal depression	
Characteristics	All children N (%)	No N (%)	Yes N (%)
**Child characteristics**			
Sex assigned at birth was male	906 (50)	826 (51)	80 (48)
Low birth weight (< 2500 grams)	184 (10)	168 (10)	16 (10)
**Paternal characteristics**			
Non-Hispanic White	433 (24)	391 (24)	42 (25)
Non-Hispanic Black	895 (50)	809 (50)	86 (52)
Hispanic	412 (23)	379 (23)	33 (20)
Other non-White	56 (3)	51 (3)	5 (3)
< High school*	556 (31)	485 (30)	71 (43)
High school graduate	604 (34)	557 (34)	47 (28)
Some college	422 (23)	387 (24)	35 (21)
College graduate	214 (12)	201 (12)	13 (8)
Foreign born*	213 (12)	206 (13)	7 (4)
< 20 years	170 (9)	151 (9)	19 (11)
20–34 years	1296 (72)	1173 (72)	123 (74)
> 34 years	330 (18)	306 (19)	24 (14)
**Family characteristics**			
Parents married*	521 (29)	490 (30)	31 (19)
Parents cohabiting	719 (40)	650 (40)	69 (42)
Neither married nor cohabiting*	556 (31)	490 (30)	66 (40)
Medicaid birth*	1053 (59)	942 (58)	111 (67)
Maternal depression at 1 year*	210 (12)	210 (12)	179 (11)
Number of observations	1,796	1,630	166

Notes: Figures are based on the sample for externalizing behaviors. All paternal and family characteristics were measured at the time of the child’s birth. *Statistically significant difference by paternal depression at 5% level using a two-tailed t-test. In addition, there were statistically significant differences by paternal depression in the percentages of fathers who were immigrants, fathers who were high school graduates, and parents who were married when the child was born, but no other characteristics, when using a Bonferroni correction for multiple comparisons.

### Statistical analysis

First, we estimated a multivariate logistic regression model predicting the child still being in the study at age 5 as a function of child, paternal, and family characteristics. Second, we documented child, paternal, and family characteristics and 1-year maternal depression in the sample, overall and by paternal depression at 1 year, and tested for statistically significant differences in each characteristic by paternal depression. Third, we documented mean scores and ranges for the children’s behavioral outcomes, both overall and by paternal depression, and tested for statistically significant differences at 5% level using two-tailed t-tests. Fourth, we estimated unadjusted and adjusted negative binomial regression models of associations between paternal depression at 1 year on the child behavior scores that differed significantly by paternal depression and report incidence rate ratios (IRRs) and 95% confidence intervals (CIs). The negative binomial functional form, a generalization of Poisson regression, is appropriate for modeling count data with a high level of dispersion. Finally, for the behavior scores that differed significantly by paternal depression, we estimated logistic regression models of high scores, defined at least 2.0 standard deviations above the sample mean, which would suggest clinical relevance.

In supplementary analyses, we alternatively used the broader measures of paternal and maternal depression described earlier; measured paternal depression at age 3 instead of age 1; classified high scores as those at least 1.5 standard deviations above the mean rather than at least 2.0 standard deviations above the mean; limited the sample to cases in which the mother was the primary caregiver at age 5; replicated the analyses using inverse probability weights to account for sample loss from the eligible sample of 3,830 cases; controlled for whether the child lived with their father more than half the time at 1 year; controlled for whether the child lived with their father more than half the time at age 5; included interactions between baseline marital status and paternal depression at 1 year; included interactions between paternal depression and child sex at birth; and included interactions between paternal and maternal depression. We also estimated models that restricted the sample to non-marital births and thus can be generalized to that policy relevant population.

Analyses were conducted using Stata Version 18.0. This study was approved by the Rutgers Biomedical and Health Sciences Institutional Review Board.

## Results

Paternal non-Hispanic White race/ethnicity was positively associated with the child still being in the study at 5 years, while the child having been classified at birth as a boy, the birth having been financed by Medicaid, and paternal foreign-born status were negatively associated with participation, suggesting that continued participation was more likely for non-minority, higher income families. No other child, father, and family characteristics had independent associations with participation (not shown). Half of the fathers in the smallest analysis sample, that for externalizing behaviors, were non-Hispanic Black, 65% had a high school education or less, 71% were unmarried, and over half (59%) had children whose deliveries were covered by Medicaid (Table 1). Almost 1 in 10 had depression during the postpartum year (166/1.796 = 9.25%).

Compared to those whose fathers did not have depression at 1 year, children whose fathers had depression at 1 year had significantly higher externalizing behaviors and attention problem scores but not internalizing behavior or social problem scores (Table 2). As such, only externalizing behaviors and attention problems were considered for subsequent analysis.

**Table 2:** Scale and sub-scale scores on the children’s behavior checklist (CBCL) at 5 years: Range in sample, cronbach’s alpha, and means for all children in the sample, children whose fathers were not depressed at 1 year, and children whose fathers were depressed at 1 year.

				Paternal depression	
	Sample	Cronbach’s	All children	No	Yes
	Range	Alpha	Mean (s.d.)	Mean (s.d.)	Mean (s.d.)
**Externalizing behaviors:**					
Aggressive	0–36	0.86	10.55*	10.33	12.72
			(6.23)	(6.13)	(6.78)
Delinquent	0–12	0.55	1.81*	1.78	2.20
			(1.65)	(1.61)	(1.98)
Total externalizing	0–45	0.87	12.37*	12.11	14.92
			(7.35)	(7.21)	(8.21)
			N = 1,796	N = 1,630	N = 166
**Internalizing behaviors:**					
Anxious	0–20	0.71	3.33	3.31	3.51
			(3.01)	(3.03)	(2.82)
Withdrawn	0–13	0.60	2.03	2.01	2.24
			(1.96)	(1.96)	(1.95)
Total internalizing	0–25	0.77	5.27	5.24	5.64
			(4.23)	(4.26)	(3.98)
			N = 1,817	N = 1,652	N = 165
**Attention Problems**	0–17	0.68	2.70*	2.65	3.24
			(2.72)	(2.69)	(3.03)
			N = 1,834	N = 1,666	N = 168
**Social Problems**	0–9	0.43	2.04	2.03	2.14
			(1.71)	(1.73)	(1.49)
			N = 1,849	N = 1,677	N = 172

Notes: s.d. = standard deviation; ranges are within our sample. N = number of observations. *Statistically significant difference by paternal depression at 5% level using a two-tailed t-test. Differences in aggressive behavior, delinquent behavior, total externalizing behavior, and attention problems by paternal depression remained statistically significant even when using a Bonferroni correction for multiple comparisons.

In unadjusted negative binomial regression models, paternal depression was associated with significantly higher externalizing behavior and attention problem scores (Table 3, panel A). The unadjusted IRR estimate of the association between paternal depression and the total externalizing subscale score was 1.23 (95% CI: 1.13–1.35); i.e., paternal depression, on average, was associated with a 23% higher score. Similarly, paternal depression was associated with a 23% higher score on the aggressive subscale, a 24% higher score on the delinquent subscale, and a 22% higher on the attention problems scale. Adjusting for child, paternal, and family characteristics and maternal depression at 1 year reduced the estimates to 17% higher score on total the externalizing subscale (IRR: 1.17; 95% CI: 1.07–1.27), 17% higher on the aggressive subscale (IRR: 1.17; 95% CI: 1.08–1.27) and 18% higher on the delinquent subscale (IRR: 1.18; 95% CI: 1.03–1.35), but paternal depression was no longer significantly associated with attention problem scores.

**Table 3:** Associations between paternal depression at 1 year and children’s externalizing behavior and attention problems at 5 years.

**Panel A**	**Negative binomial regression estimates**			
	**Aggressive IRR (95% CI)**	**Delinquent IRR (95% CI)**	**Total externalizing IRR (95% CI)**	**Attention Problems IRR (95% CI)**
Unadjusted	1.23 (1.13–1.34)	1.24 (1.07–1.43)	1.23 (1.13–1.35)	1.22 (1.05–1.42)
Adjusted for child, paternal, and family characteristics	1.19 (1.09–1.30)	1.20 (1.04–1.38)	1.19 (1.09–1.30)	1.17 (1.01–1.36)
Adjusted for child characteristics, paternal characteristics, family characteristics, and maternal depression	1.17 (1.08–1.27)	1.18 (1.03–1.35)	1.17 (1.07–1.27)	1.12 (0.97–1.29)
	N = 1,796	N = 1,796	N = 1,796	N = 1,834
**Panel B**	**Adjusted logistic regression estimates**			
	**Aggressive OR (95% CI)**	**Delinquent OR (95% CI)**	**Total externalizing OR (95% CI)**	**Attention Problems OR (95% CI)**
Unadjusted	2.90 (1.60–5.26)	2.31 (1.14–4.68)	3.57 (2.07–6.16)	1.94 (1.05–3.59)
Adjusted for child, paternal, and family characteristics	2.49 (1.34–4.60)	2.11 (1.01–4.40)	3.20 (1.81–5.64)	1.81 (0.95–3.45)
Adjusted for child characteristics, paternal characteristics, family characteristics, and maternal depression	2.40 (1.30–4.43)	2.08 (1.01–4.27)	3.09 (1.77–5.41)	1.72 (0.91–3.26)
	N = 1,796	N = 1,796	N = 1,796	N = 1,834

Notes: IRR = incidence rate ratios. OR = odds ratio. CI = confidence interval. N = number of observations. All adjusted models control for child, paternal, and family characteristics in Table 2. Outcome in logistic regressions is a high score, defined as at least 2.0 standard deviation above the sample mean.

Controlling for paternal depression and all other covariates, children whose parents were married when they were born had significantly lower aggressive, delinquent, total externalizing, and attention problem scores compared to children born to unmarried parents (S2 Table). Children whose fathers had at least some college education scored significantly lower on the aggressive, total externalizing, and attention problem scales compared to children whose fathers had less than a high school degree. Children whose fathers had a college degree also scored lower than those whose fathers had less than a high school education on the delinquent scores. Medicaid births were associated with higher scores on all but the delinquent scale and maternal depression was associated with higher scores on all four scales. Children assigned at birth as males had uniformly higher externalizing behavior and attention scores compared to those assigned at birth as females.

In unadjusted logistic regression models of high externalizing behavior scores (at least 2.0 standard deviations above the mean), paternal depression was associated with about 2–3.5 times the odds of high aggressive, delinquent, and total externalizing behavior scores and high attention problem scores (Odds Ratio (OR): 2.90; 95% CI: 1.60–5.26; OR: 2.31; 95% CI: 1.14–4.68; OR: 3.57; 95% CI: 2.07–6.16; and OR: 1.94; 95% CI: 1.05–3.59, respectively; Table 3, panel B) compared to adolescents whose fathers did not have depression. When controlling for child, paternal, family characteristics and maternal depression, the magnitudes were somewhat smaller than in the unadjusted models, but paternal depression was still associated with 2–3 times the odds of high aggressive, delinquent, and total externalizing behavior scores (OR: 2.40; 95% CI: 1.30–4.43; OR: 2.08; 95% CI: 1.01–4.27; and OR: 3.09; 95% CI: 1.77–5.41, respectively).

## Discussion

In a relatively disadvantaged U.S. population-based birth cohort, about 9% of the fathers had depressive symptoms in the postpartum year, which—despite the unique population—is consistent with the national and international literature [44]. Paternal depression was associated with 17–18% higher scores for children’s externalizing behaviors, including aggressive and delinquent behaviors, at age 5, even when controlling for child, paternal, and family characteristics, as well as maternal depression during the postpartum year. Paternal depression was also associated with total externalizing behavior scores that were at least 2 standard deviations above the sample mean, which suggests clinical relevance. Specifically, in logistic regression models that controlled for child, paternal, and family characteristics and maternal depression at 1 year, paternal depression was associated with 2–3 times the odds of high aggressive behavior scores, high delinquency scores, and high total externalizing scores. We focused on a population that included a large number and share of children with unmarried parents and non-resident fathers, a population in the U.S. that is sizable, disproportionately disadvantaged, and under-researched in this context [11].

We linked paternal depression to subsequent child outcomes, which alleviates concerns about potential reverse causality—i.e., children’s behavioral problems leading to paternal depression, as has been found in previous research [45]. In our main analyses, we did not adjust for paternal residence in the child’s household at the time of the child’s behavioral assessment because studies have found that maternal depression is associated with subsequent parental relationship dissolution [46], and the same could be true for paternal depression. In that case, the father’s presence in the household could be an outcome of the depression and lie on the pathway between paternal depression and children’s behavioral outcomes.

In the U.S., nationally representative studies of 2-parent families have found cross-sectional associations between paternal depression and 5–17 year old children’s behavioral problems [7], and between paternal depression or anxiety and 3–12 year old children’s internalizing behaviors but not externalizing behaviors, adjusting for maternal depression [6]. Thus, the studies focused on very large age ranges of children and estimated concurrent associations between paternal depression and children’s behaviors, and neither included non-residential fathers.

Studies of disadvantaged U.S. populations that included non-residential fathers found that: (1) paternal depression at age 3 appeared to partially explain associations between multiple-partner fertility and child outcomes at age 3, including externalizing behaviors [23], (2) there were no cross-sectional associations between paternal depression or anxiety and children’s anxiety or depression, attention deficit, or oppositional defiant disorders at age 3 [47], (3) there were significant associations between paternal depression when children were 2 years old and internalizing behaviors at age 5, but not externalizing behaviors [48], and (4) paternal depression when children were 2–3 years old was associated with lower levels of self-control and cooperation and higher levels of hyperactivity on teacher-reported social skills at 5^th^ grade, controlling for maternal depression and other child and family characteristics [49]. Our findings address gaps left by these studies, which focused on paternal depression or anxiety [47] or depression [23] concurrently with children’s externalizing behaviors at age 3 [23, 47], did not control for maternal depression [23], or used small, specialized samples that did not allow for generality [48, 49].

Healthy social-emotional functioning at age 5 is important for kindergarten readiness and sets the stage for future emotional, school, and employment success [50]. Externalizing behaviors and inattention and/or hyperactivity are associated with poor school performance [51–53], risky and delinquent behaviors [35, 50, 54–56], poor peer interactions [53], and later development of psychopathology [51, 53, 57]. Children’s adverse social-emotional functioning is also associated with increased family stress [58] and poorer parenting interactions, including harsh punishment [59, 60]. The postpartum year is an important exposure period because it forms the foundation for parenting behaviors [61, 62], father involvement [63], interactions leading to healthy attachment [64], and child development [65]. Transitioning to parenthood is also strongly associated with the mental health of parents, including fathers [13, 14, 29, 66].

Paternal depression could affect children’s behaviors through various channels. Studies have found that depressed fathers are more likely to physically punish their children [60, 61] and engage in harsh and controlling parenting [3, 67], both of which are associated with children’s externalizing behaviors and attention problems [59, 68, 69]. There is some evidence that fathers with depression are less likely to be strongly attached to their infants and emotionally supportive of their children [64, 67, 70, 71] and to engage in positive parenting practices including shared reading [60], which has been associated with less harsh parenting [72]. Paternal depression may lead to marital conflict [3] and poorer parenting or co-parenting, which could lead the father to spend less time in the child’s household [71], although our estimates did not change when we controlled for the father living in the household at least half the time. Finally, there is some evidence that parental depression can impact externalizing behavior via both genetic and environmental pathways [73]. Many of these mechanisms can be explored in future research.

Our findings support the need to better understand the impacts of paternal depression and expand early identification and intervention of depression in fathers to promote optimal developmental and behavioral health in children. Screening and identification of paternal depression in pediatric and other health care practices in the U.S. is rare [13, 74]. Given their near-universal access to children, frequent contact with families through recommended well visits, and opportunities to build on established relationships, pediatricians are well-positioned to identify parents at risk for depression [75]. Integration of maternal depression screening and intervention is widely supported in health care and public policy. Expansion to include fathers, coupled with better research and development of father-focused interventions could help promote child and family well-being. Our work highlights the importance of including nonresidential and unmarried fathers and under-represented populations in this work. Further research into paternal depression beyond the postpartum period and its impact on child outcomes is also needed.

Strengths of our study include the generally disadvantaged population-based U.S. sample, measurement of paternal depression and children’s outcomes at developmentally important time points, the prospective linkage of paternal depression to subsequent child outcomes, the inclusion of rich control variables including maternal depression; and use of validated scales to assess both depression and children’s behaviors. Another strength was having paternal assessments of depression based on fathers’ survey responses and assessments of child behavior from primary caregivers. Studies have found that fathers with depression are more likely than their children’s mothers and teachers to report children’s externalizing behaviors [3].

Limitations include loss to follow-up. Children of more disadvantaged fathers were less likely to be followed up at age 5, which could bias the estimates although the expected direction of bias is not clear. In addition, although we were able to prospectively follow the families and control for rich covariates including, maternal depression during the postpartum year, causality cannot be firmly established in any observational study. While our study did not find that paternal depression was significantly associated with internalizing behaviors of children at 5 years, internalizing behaviors often do not become apparent during the pre-school years [76, 77]; future work focusing on older children should further examine internalizing behaviors. Chronbach’s alpha for the delinquent subscale and children’s social problems were low, so findings regarding those outcomes may not be reliable. We could not account for the intensity or duration of the child’s exposure to paternal depression. Information about the gender of the child was not collected in the FFCWB study until the children were 15 years old.

The findings cannot necessarily be generalized to countries other than the U.S., and the focus was on a cohort that was born 23–25 years ago so we cannot be sure that the findings would apply to more recent U.S. cohorts. Supports and resources for parents, especially fathers, have contracted in the U.S. since that time, so it is possible that the associations have gotten larger. We know of no more recent population-based data (U.S. or otherwise) that prospectively followed fathers as well as their children from birth to age 5 with fathers’ screenings for depression and their children’s behaviors assessed by primary caregivers (independent reporters) using validated instruments. Thus, the data we used are the most recent and most appropriate for studying linkages between paternal depression and children’s behavior in a population-based U.S. sample. Prospective data collection on a new cohort with similar measures would provide a potentially fruitful direction for future research. Finally, the cohort we studied is of interest since those children are now having children of their own and their life course development has implications for the next generation.

In summary, this study found strong associations between paternal depression in the first year of life and externalizing behaviors and attention problems at age 5 when children typically transition into school. These findings support the need to identify fathers at risk for depression and link them to interventions that can support optimal child development and well-being. Given their frequent and near-universal contact with families, pediatricians are well-positioned to address this important need.

## Supporting information

S1 File Supplementary results. (DOCX)

S1 Table Components of aggressive, delinquent behavior, anxious, withdrawn, social problems and attention scores from the child behavior checklist. Notes: Total Externalizing (34 items) = Aggressive + Delinquent; Total Internalizing (22 items) = Anxious +Withdrawn. One item (unhappy, sad, or depressed) is included in both and only counts once toward internalizing.
(DOCX)

S2 Table Adjusted negative binomial regression estimates of associations between paternal depression at 1 year and children’s externalizing behaviors and attention problems at 5 years. Notes: IRR = incidence rate ratios. CI = confidence interval. N = number of observations. Estimates from Panel A of Table 3, fully-adjusted models.
(DOCX)

S3 Table Associations between paternal depression at 1 year and children’s externalizing behavior and attention problems at 5 years, using inverse probability weights. Notes: IRR = incidence rate ratios. AOR = adjusted odds ratio. CI = confidence interval. All adjusted models control for child, paternal, and family characteristics in Table 2. Outcome in logistic regressions is a high score, defined as > = 2.0 standard deviation above the sample mean.
(DOCX)

S4 Table Associations between paternal depression at 1 year and children’s externalizing behavior and attention problems at 5 years, restricting the sample to non-marital births. Notes: IRR = incidence rate ratios. AOR = adjusted odds ratio. CI = confidence interval. All adjusted models control for child, paternal, and family characteristics in Table 2. Outcome in logistic regressions is a high score, defined as > = 2.0 standard deviation above the sample mean.
(DOCX)
