# Supplementary material for: Paternal depression in the postpartum year and children’s behaviors at age 5 in an urban U.S. birth cohort
Source: PLoS One. 2024 Apr 18;19(4):e0300018. doi: 10.1371/journal.pone.0300018 (PMC11025738; doi:10.1371/journal.pone.0300018)
Supplement: S1 File — (DOCX) [file pone.0300018.s001.docx]

Supplementary results (not shown unless indicated otherwise)

Supplementary models that used the broader measure of paternal and maternal depression; measured paternal depression at age 3 instead of age 1; used the less stringent measure of high scores (at least 1.5 standard deviations above the mean); limited the sample to cases in which the mother was the primary caregiver at age 5; and replicated the analyses using inverse probability weights (S3 table) to account for sample loss all produced estimates similar to those in Table 3. Additionally, controlling for whether the father lived with the child for more than half the time when the child was age 1 also produced estimates similar to those in Table 3, as did controlling for whether the father lived with the child more than half the time at age 5.

Although children with married fathers had lower scores on all externalizing behavior outcomes and attention problems (S2 Table), there were no significant interactive associations between paternal depression and baseline marital status. Similarly, although children whose sex at birth was male had higher externalizing behavior scores than those whose sex at birth was female, there were no significant interactions between paternal depression and child sex at birth. Paternal depression and maternal depression had significant interactive associations with delinquent behaviors, but not with aggressive or total externalizing behaviors. That is, for delinquent behaviors, both parents being depressed was associated with significantly worse behaviors beyond the independent associations of each parent being depressed.

Finally, in models that restricted the sample to non-marital births, the estimates from the negative binomial regressions and logistic regression analyses were very similar to those in Table 3 (S4 Table). All associations between paternal depression and behavioral outcomes remained statistically significant, with one exception—paternal depression was no longer a significant predictor of high delinquent behavior scores.
