# Supplementary material for: Paternal depression in the postpartum year and children’s behaviors at age 5 in an urban U.S. birth cohort
Source: PLoS One. 2024 Apr 18;19(4):e0300018. doi: 10.1371/journal.pone.0300018 (PMC11025738; doi:10.1371/journal.pone.0300018)
Supplement: S1 Table — Notes: Total Externalizing (34 items) = Aggressive + Delinquent; Total Internalizing (22 items) = Anxious +Withdrawn. One item (unhappy, sad, or depressed) is included in both and only counts once toward internalizing. (DOCX) [file pone.0300018.s002.docx]

**S1 Table: Components of aggressive, delinquent behavior, anxious, withdrawn, social problems and attention scores from the Child Behavior Checklist**

| **Aggressive (20 items)** |
| --- |
| Argues a lot  Brags or boasts  Cruel, bullying or mean to others  Demands a lot of attention  Destroys his/her own things  Destroys things belonging to family/others  Disobedient at home  Disobedient in school  Easily jealous  Gets in many fights  Physically attacks people  Screams a lot  Showing off/clowning  Stubborn/sullen/irritable  Has sudden changes in mood or feelings  Talks too much  Teases a lot  Has temper tantrums or hot temper  Threatens people  Unusually loud |
| **Delinquent Behavior (10 items)** |
| Doesn’t seem to feel guilt after misbehaving  Hangs around w/ others who get in trouble  Lies or cheats  Prefers being with older kids  Runs away from home  Sets fire  Steals at home  Steals outside home  Swears or uses obscene language  Vandalizes |
| **Anxious (14 items)**  Complains of loneliness  Cries a lot  Fears s/he might think/do something wrong  Feels s/he has to be perfect  Feels/complains no one loves him/her  Feels others out to get him/her  Feels worthless/inferior  Nervous, high strung or tense  Too fearful or anxious  Feels too guilty  Self-conscious or easily embarrassed  Suspicious  Unhappy, sad or depressed  Worries |
| **Withdrawn (9 items)** |
| Would rather be alone than with others  Refuses to talk  Secretive, keeps things to self  Shy or timid  Stares blankly  Sulks a lot  Underactive, slow moving, lacks energy  Unhappy, sad, or depressed  Withdrawn, doesn’t get involved with others |
| **Attention Problems (11 items)** |
| Acts too young for age  Can’t concentrate  Can’t sit still  Confused or seems to be in a fog  Daydreams or gets lost in his/her thoughts  Impulsive or acts without thinking  Nervous, high-strung or tense  Nervous moment or twitching  Has poor school work  Poorly coordinated or clumsy  Stares blankly |
| **Social Problems (8 items)** |
| Acts too young for age  Clings to adults or too dependent  Does not get along with other kids  Gets teased a lot  Not liked by other kids  Overweight  Poorly coordinated or clumsy  Prefers being with younger kids |

Notes:

Total Externalizing (34 items) = Aggressive + Delinquent

Total Internalizing (22 items) = Anxious +Withdrawn. One item (unhappy, sad, or depressed) is included in both and only counts once toward internalizing.
