# Supplementary material for: Paternal depression in the postpartum year and children’s behaviors at age 5 in an urban U.S. birth cohort
Source: PLoS One. 2024 Apr 18;19(4):e0300018. doi: 10.1371/journal.pone.0300018 (PMC11025738; doi:10.1371/journal.pone.0300018)
Supplement: S2 Table — Notes: IRR = incidence rate ratios. CI = confidence interval. N = number of observations. Estimates from Panel A of Table 3, fully-adjusted models. (DOCX) [file pone.0300018.s003.docx]

**S2 Table: Adjusted negative binomial regression estimates of associations between paternal depression at 1 year and children’s externalizing behaviors and attention problems at 5 years.**

|  | **Aggressive**  **IRR**  **(95% CI)** | **Delinquent**  **IRR**  **(95% CI)** | **Total Externalizing**  **IRR**  **(95% CI)** | **Attention Problems**  **IRR**  **(95% CI)** |
| --- | --- | --- | --- | --- |
| Paternal depression at 1 year | 1.17  (1.08 - 1.27) | 1.18  (1.03 - 1.35) | 1.17  (1.07 - 1.27) | 1.12  (0.97 - 1.29) |
| Child characteristics |  |  |  |  |
| Sex assigned at birth was male | 1.08  (1.03 – 1.14) | 1.21  (1.11 – 1.31) | 1.10  (1.04 – 1.16) | 1.27  (1.16 - 1.39) |
| Low birth weight | 1.04  (0.95 – 1.14) | 1.02  (0.89 – 1.16) | 1.04  (0.95 – 1.13) | 1.09  (0.93 - 1.28) |
| Paternal characteristics |  |  |  |  |
| Non-Hispanic Black | 0.96  (0.89 – 1.06) | 1.10  (0.98 – 1.23) | 0.98  (0.91 – 1.05) | 0.73  (0.64 - 0.83) |
| Hispanic | 0.97  (0.89 – 1.06) | 1.03  (0.90 – 1.18) | 0.98  (0.90 – 1.07) | 0.85  (0.74 - 0.98) |
| Other non-White | 0.99  (0.85 – 1.15) | 0.99  (0.76 – 1.29) | 0.99  (0.85 – 1.15) | 0.73  (0.56 - 0.95) |
| High school graduate | 0.93  (0.87 – 0.99) | 0.97  (0.88 – 1.08) | 0.93  (0.87 – 1.00) | 0.92  (0.82 - 1.02) |
| Some college | 0.90  (0.83 – 0.97) | 0.92  (0.82 – 1.03) | 0.90  (0.84 – 0.98) | 0.88  (0.77 - 1.00) |
| College graduate | 0.82  (0.74 – 0.91) | 0.77  (0.65 – 0.92) | 0.81  (0.73 – 0.90) | 0.65  (0.53 - 0.79) |
| Foreign born | 0.97  (0.88 – 1.06) | 1.01  (0.88 – 1.17) | 0.97  (0.89 – 1.06) | 1.07  (0.92 - 1.25) |
| < 20 years | 1.07  (0.95 – 1.21) | 1.11  (0.93 – 1.32) | 1.08  (0.96 – 1.21) | 1.17  (0.96 - 1.42) |
| 20–34 years | 1.05  (0.97 – 1.13) | 1.04  (0.93 – 1.16) | 1.05  (0.97 – 1.13) | 1.11  (0.97 - 1.27) |
| Family characteristics |  |  |  |  |
| Married | 0.90  (0.82 – 0.98) | 0.85  (0.75 – 0.97) | 0.89  (0.82 – 0.97) | 0.86  (0.74 - 0.99) |
| Cohabiting | 0.98  (0.91 – 1.04) | 0.94  (0.85 – 1.04) | 0.97  (0.91 – 1.04) | 0.89  (0.80 - 0.99) |
| Medicaid birth | 1.07  (1.01 – 1.14) | 1.10  (1.00 – 1.20) | 1.08  (1.01 – 1.15) | 1.17  (1.05 - 1.30) |
| Maternal depression at 1 year | 1.23  (1.14 – 1.32) | 1.18  (1.04 – 1.32) | 1.22  (1.13 – 1.31) | 1.44  (1.28 - 1.63) |
|  | N = 1,796 | N = 1,796 | N = 1,796 | N = 1,834 |

Notes: IRR = incidence rate ratios. CI = confidence interval. N = number of observations. Estimates from Panel A of Table 3, fully-adjusted models
