# Supplementary material for: Paternal depression in the postpartum year and children’s behaviors at age 5 in an urban U.S. birth cohort
Source: PLoS One. 2024 Apr 18;19(4):e0300018. doi: 10.1371/journal.pone.0300018 (PMC11025738; doi:10.1371/journal.pone.0300018)
Supplement: S3 Table — Notes: IRR = incidence rate ratios. AOR = adjusted odds ratio. CI = confidence interval. All adjusted models control for child, paternal, and family characteristics in Table 2. Outcome in logistic regressions is a high score, defined as > = 2.0 standard deviation above the sample mean. (DOCX) [file pone.0300018.s004.docx]

**S3 Table: Associations between paternal depression at 1 year and children’s externalizing behavior and attention problems at 5 years, using inverse probability weights**

| **Panel A** | **Negative binomial regression estimates** | | | |
| --- | --- | --- | --- | --- |
|  | **Aggressive**  **IRR**  **(95% CI)** | **Delinquent**  **IRR**  **(95% CI)** | **Total externalizing**  **IRR**  **(95% CI)** | **Attention Problems**  **IRR**  **(95% CI)** |
| Unadjusted | 1.24  (1.14 - 1.35) | 1.24  (1.07 - 1.43) | 1.24  (1.13 - 1.35) | 1.21  (1.04 - 1.42) |
| Adjusted for child, paternal, and family characteristics | 1.20  (1.10 - 1.31) | 1.20  (1.04 - 1.38) | 1.20  (1.10 - 1.31) | 1.17  (1.00 - 1.36) |
| Adjusted for child characteristics, paternal characteristics, family characteristics, and maternal depression | 1.18  (1.08 - 1.28) | 1.18  (1.02 - 1.35) | 1.18  (1.08 - 1.28) | 1.11  (0.96 - 1.29) |
|  | N = 1,796 | N = 1,796 | N = 1,796 | N = 1,834 |
|  |  |  |  |  |
| **Panel B** | **Adjusted logistic regression estimates** | | | |
|  | Aggressive  AOR  (95% CI) | Delinquent  AOR  (95% CI) | Total externalizing  AOR  (95% CI) | Attention Problems  AOR  (95% CI) |
| Unadjusted | 2.95  (1.61 - 5.39) | 2.16  (1.06 - 4.42) | 3.49  (2.01 - 6.08) | 2.08  (1.11 - 3.89) |
| Adjusted for child, paternal, and family characteristics | 2.57  (1.39 - 4.76) | 1.99  (0.95 - 4.18) | 3.18  (1.80 - 5.62) | 2.01  (1.04 - 3.88) |
| Adjusted for child characteristics, paternal characteristics, family characteristics, and maternal depression | 2.46  (1.33 - 4.54) | 1.95  (0.94 - 4.04) | 3.05  (1.74 - 5.36) | 1.91  (1.00 - 3.64) |
|  | N = 1,796 | N = 1,796 | N = 1,796 | N = 1,834 |

Notes: IRR = incidence rate ratios. AOR = adjusted odds ratio. CI = confidence interval. All adjusted models control for child, paternal, and family characteristics in Table 2. Outcome in logistic regressions is a high score, defined as >= 2.0 standard deviation above the sample mean.
