# Supplementary material for: Paternal depression in the postpartum year and children’s behaviors at age 5 in an urban U.S. birth cohort
Source: PLoS One. 2024 Apr 18;19(4):e0300018. doi: 10.1371/journal.pone.0300018 (PMC11025738; doi:10.1371/journal.pone.0300018)
Supplement: S4 Table — Notes: IRR = incidence rate ratios. AOR = adjusted odds ratio. CI = confidence interval. All adjusted models control for child, paternal, and family characteristics in Table 2. Outcome in logistic regressions is a high score, defined as > = 2.0 standard deviation above the sample mean. (DOCX) [file pone.0300018.s005.docx]

**S4 Table: Associations between paternal depression at 1 year and children’s externalizing behavior and attention problems at 5 years, restricting the sample to non-marital births**

| **Panel A** | **Negative binomial regression estimates** | | | |
| --- | --- | --- | --- | --- |
|  | **Aggressive**  **IRR**  **(95% CI)** | **Delinquent**  **IRR**  **(95% CI)** | **Total externalizing**  **IRR**  **(95% CI)** | **Attention Problems**  **IRR**  **(95% CI)** |
| Unadjusted | 1.21  (1.11 - 1.33) | 1.22  (1.04 - 1.42) | 1.21  (1.10 - 1.34) | 1.13  (0.96 - 1.33) |
| Adjusted for child, paternal, and family characteristics | 1.20  (1.09 - 1.32) | 1.21  (1.04 - 1.41) | 1.20  (1.09 - 1.33) | 1.12  (0.96 - 1.32) |
| Adjusted for child characteristics, paternal characteristics, family characteristics, and maternal depression | 1.19  (1.08 - 1.31) | 1.20  (1.03 - 1.40) | 1.19  (1.08 - 1.31) | 1.08  (0.93 - 1.26) |
|  | N = 1,275 | N = 1,275 | N = 1,275 | N = 1,296 |
|  |  |  |  |  |
| **Panel B** | **Adjusted logistic regression estimates** | | | |
|  | **Aggressive**  **AOR**  **(95% CI)** | **Delinquent**  **AOR**  **(95% CI)** | **Total externalizing**  **AOR**  **(95% CI)** | **Attention Problems**  **AOR**  **(95% CI)** |
| Unadjusted | 2.63  (1.41 - 4.92) | 2.02  (0.95 - 4.26) | 3.08  (1.72 - 5.50) | 1.75  (0.89 - 3.44) |
| Adjusted for child, paternal, and family characteristics | 2.55  (1.33 - 4.87) | 2.03  (0.94 - 4.38) | 3.05  (1.68 - 5.56) | 1.75  (0.87 - 3.55) |
| Adjusted for child characteristics, paternal characteristics, family characteristics, and maternal depression | 2.50  (1.31 - 4.77) | 2.00  (0.94 - 4.27) | 2.98  (1.64 - 5.40) | 1.69  (0.84 - 3.37) |
|  | N = 1,275 | N = 1,275 | N = 1,275 | N = 1,296 |

Notes: IRR = incidence rate ratios. AOR = adjusted odds ratio. CI = confidence interval. All adjusted models control for child, paternal, and family characteristics in Table 2. Outcome in logistic regressions is a high score, defined as >= 2.0 standard deviation above the sample mean.
